# Supplementary material for: Analysis of Stored Wheat Grain-Associated Microbiota Reveals Biocontrol Activity among Microorganisms against Mycotoxigenic Fungi
Source: J Fungi (Basel). 2021 Sep 20;7(9):781. doi: 10.3390/jof7090781 (PMC8470753; doi:10.3390/jof7090781)
Supplement: Supplementary file 1 [file jof-07-00781-s001.zip › jof-1375842-supplementary.pdf]

Supplementary Material

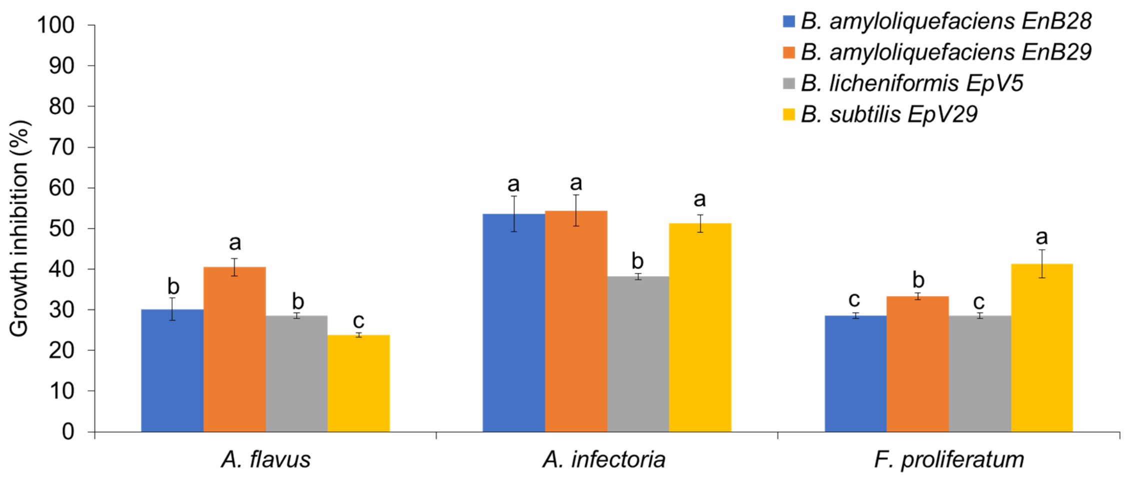

Figure S1. Graphical representation of antagonistic activity of four selected bacterial isolates against three mycotoxigenic fungal pathogens *in vitro*. Data are means of at least three independent repetitions ± standard error. One-way ANOVA differences were considered significant when $p < 0.05$. Different letters above the error bars indicate statistically significant differences among treatments in each group as determined based on Duncan's multiple range test.

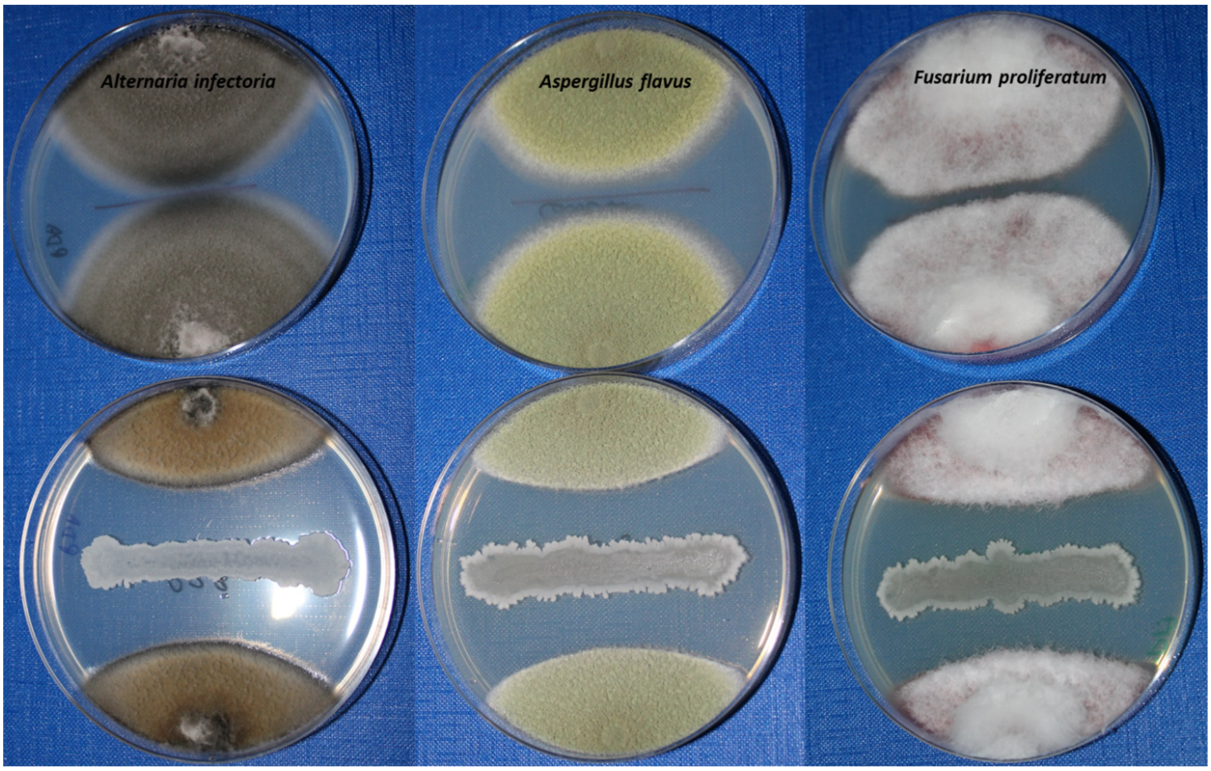

Figure S2. Antagonistic activity of *Bacillus amyloliquefaciens* (strain EnB28) against three mycotoxigenic fungal pathogens *in vitro*.

Table S1. List of primers used in the study

Target	Primer name	Sequence (5′-3′)	Reference	PCR conditions
[a]16S rRNA	799F	GAAGTAAAAGTCGTAACAAGG*AACMGGATTAGATACCCKG*	[1]	95°C-2 min; 28 cycles (94°C-45s, 49°C-45s & 72°C-60s); 72°C-10 min
	1392R	*GTCTCGTGGGCTCGGAGATGTGTATAAGAGACAG*ACGGGCGGTGTGTRC		
[a]ITS	ITS 5F	*TCGTCGGCAGCGTCAGATGTGTATAAGAGACAG*GGAAGTAAAAGTCGTAACAAGG	[2]	95°C-2 min; 25 cycles (95°C-30s, 50°C-30s & 72°C-30s); 72°C-10 min
	ITS 86R	*GTCTCGTGGGCTCGGAGATGTGTATAAGAGACAG*TTCAAAGATTCGATGATTCAC		
[b]16S rRNA	27F	AGAGTTTGATCMTGGCTCAG	[3]	95°C-10 min, 35 cycles (90°C-30s, 50°C-30s & 72°C-2 min); 72°C-5 min
	1492R	TACGGYTACCTTGTTACGACTT		
[b]ITS	ITS1	TCCGTAGGTGAACCTGCGG	[4]	95°C-3 min, 30 cycles (95°C-30s, 56°C-60s & 72°C-90s); 72°C-5 min
	ITS4	TCCTCCGCTTATTGATATGC		
[b]D1/D2 domains of the LSU	NL1	GCATATCAATAAGCGGAGGAAAAG	[5]	94°C-4 min; 45 cycles (94°C-20s, 53°C -20s and 72°C-90s); 72°C-5 min
	NL4	GGTCCGTGTTTCAAGACGG		

[a] Illumina MiSeq sequencing (adapter sequences are italicized)

[b] Sanger sequence

References

1. Hanshew, A.S.; Mason, C.J.; Raffa, K.F.; Currie, C.R. Minimization of chloroplast contamination in 16S rRNA gene pyrosequencing of insect herbivore bacterial communities. *J. Microbiol. Methods* **2013**, *95*, 149–155, doi:10.1016/j.mimet.2013.08.007.

2. Scibetta, S.; Schena, L.; Abdelfattah, A.; Pangallo, S.; Cacciola, S.O. Selection and experimental evaluation of universal primers to study the fungal microbiome of higher plants. *Phytobiomes J.* **2018**, *2*, 225–236, doi:10.1094/PBIOMES-02-18-0009-R.

3. Weisburg, W.G.; Barns, S.M.; Pelletier, D.A.; Lane, D.J. 16S ribosomal DNA amplification for phylogenetic study. *J. Bacteriol.* **1991**, *173*, 697–703.

4. Mitchell, T.G.; Freedman, E.Z.; White, T.J.; Taylor3, J.W. Unique oligonucleotide primers in PCR for identification of *Cryptococcus neoformans*. *J. Clin. Microbiol.* **1994**, 253–255.

5. Dagar, S.S.; Kumar, S.; Mudgil, P.; Singh, R.; Puniya, A.K. D1/D2 domain of large-subunit ribosomal DNA for differentiation of Orpinomyces spp. *Appl. Environ. Microbiol.* **2011**, *77*, 6722–6725, doi:10.1128/AEM.05441-11.

Table S2. List of bacterial taxa with significant differential abundance according to Kruskal-Wallis test

Taxa	Test-Statistic	P	FDR_P	Bonferroni_P	Endophytes_ mean	Epiphytes_ mean
D_0__Bacteria;D_1__Firmicutes;D_2__Clostridia;D_3__Clostridiales ;D_4__Ruminococcaceae;Other	41.32928475	1.29E-10	6.95E-09	6.95E-09	0.020538224	8.83E-05
D_0__Bacteria;D_1__Planctomycetes;D_2__Phycisphaerae;D_3__ MSBL9;D_4__SG8-4;Other	38.58710343	5.24E-10	1.41E-08	2.83E-08	0.037178975	8.08E-05
D_0__Bacteria;D_1__Chloroflexi;D_2__Anaerolineae;D_3__Anaerol ineales;D_4__Anaerolineaceae;Other	37.33109636	9.97E-10	1.41E-08	5.38E-08	0.015681998	0.000169697
D_0__Bacteria;D_1__Proteobacteria;D_2__Alphaproteobacteria;D_ 3__Rhizobiales;D_4__Devosiaceae;D_5__Devosia	36.9266055	1.23E-09	1.41E-08	6.62E-08	0.00401888	0
D_0__Bacteria;D_1__Actinobacteria;D_2__Actinobacteria;Other;Ot her;Other	33.51515558	7.07E-09	5.73E-08	3.82E-07	0.020745893	0.000337869
D_0__Bacteria;Other;Other;Other;Other;Other	33.41783805	7.43E-09	5.73E-08	4.01E-07	0.01250916	8.83E-05
D_0__Bacteria;D_1__Actinobacteria;D_2__Actinobacteria;D_3__Mi crococcales;D_4__Microbacteriaceae;Other	33.04153952	9.02E-09	6.09E-08	4.87E-07	0.001190341	0.06100626
D_0__Bacteria;D_1__Proteobacteria;D_2__Alphaproteobacteria;D_ 3__Sphingomonadales;D_4__Sphingomonadaceae;D_5__Sphingom onas	28.31148822	1.03E-07	6.20E-07	5.58E-06	0.004636835	0.119954212
D_0__Bacteria;D_1__Actinobacteria;D_2__Actinobacteria;D_3__Mi crococcales;D_4__Microbacteriaceae;D_5__Agrococcus	26.34504507	2.86E-07	1.54E-06	1.54E-05	0.000471075	0.032707256
D_0__Bacteria;D_1__Actinobacteria;D_2__Actinobacteria;D_3__Mi crococcales;D_4__Microbacteriaceae;D_5__Curtobacterium	24.06802757	9.30E-07	4.57E-06	5.02E-05	0.003180241	0.052611617
D_0__Bacteria;D_1__Proteobacteria;D_2__Gammaproteobacteria; D_3__Diplorickettsiales;D_4__Diplorickettsiaceae;Other	21.96078431	2.78E-06	1.25E-05	0.000150271	0.003250498	0
D_0__Bacteria;D_1__Proteobacteria;D_2__Gammaproteobacteria; D_3__Betaproteobacteriales;D_4__Burkholderiaceae;D_5__Massili a	21.79545672	3.03E-06	1.26E-05	0.000163791	0.002388715	0.066053411
D_0__Bacteria;D_1__Proteobacteria;D_2__Gammaproteobacteria; D_3__Alteromonadales;D_4__Pseudoalteromonadaceae;D_5__Pse udoalteromonas	21.23044248	4.07E-06	1.57E-05	0.000219912	0.004665161	0.00011396
D_0__Bacteria;D_1__Bacteroidetes;D_2__Bacteroidia;D_3__Cytoph agales;D_4__Hymenobacteraceae;D_5__Hymenobacter	20.91057207	4.81E-06	1.73E-05	0.000259865	0.000405013	0.009259168
D_0__Bacteria;D_1__Proteobacteria;D_2__Alphaproteobacteria;D_ 3__Rhizobiales;D_4__Beijerinckiaceae;D_5__Methylobacterium	20.32756625	6.53E-06	2.20E-05	0.00035237	0.00023363	0.00928243
D_0__Bacteria;D_1__Proteobacteria;D_2__Alphaproteobacteria;D_ 3__Rhodobacterales;D_4__Rhodobacteraceae;D_5__Paracoccus	20.09112069	7.38E-06	2.35E-05	0.000398729	0.000878895	0.034654902

D_0__Bacteria;D_1__Proteobacteria;D_2__Gammaproteobacteria;D_3__Pseudomonadales;D_4__Pseudomonadaceae;D_5__Pseudomonas	19.66015273	9.25E-06	2.75E-05	0.000499557	0.011806024	0.087823211
D_0__Bacteria;D_1__Bacteroidetes;D_2__Bacteroidia;D_3__Cytophagales;D_4__Microscillaceae;D_5__Ohtaekwangia	19.57556768	9.67E-06	2.75E-05	0.000522169	0.008461623	8.89E-05
D_0__Bacteria;D_1__Actinobacteria;D_2__Actinobacteria;D_3__Micrococcales;D_4__Microbacteriaceae;D_5__Clavibacter	16.64368978	4.51E-05	0.00012177	0.002435405	0.000116544	0.004258944
D_0__Bacteria;D_1__Proteobacteria;D_2__Gammaproteobacteria;D_3__Enterobacteriales;D_4__Enterobacteriaceae;D_5__Pantoea	16.36602353	5.22E-05	0.000134262	0.002819494	0.077038506	0.246165732
D_0__Bacteria;D_1__Proteobacteria;D_2__Gammaproteobacteria;D_3__Enterobacteriales;D_4__Enterobacteriaceae;Other	15.3727483	8.83E-05	0.000216618	0.004765606	0.000429613	0.01308082
D_0__Bacteria;D_1__Actinobacteria;D_2__Actinobacteria;D_3__Micrococcales;D_4__Micrococcaceae;D_5__Arthrobacter	15.17919188	9.78E-05	0.000229559	0.005279846	0.000173947	0.006100557
D_0__Bacteria;D_1__Proteobacteria;D_2__Gammaproteobacteria;D_3__Betaproteobacteriales;D_4__Burkholderiaceae;D_5__Comamonas	13.54271357	0.000233195	0.000524689	0.012592529	0.00597489	0
D_0__Bacteria;D_1__Firmicutes;D_2__Bacilli;D_3__Bacillales;D_4__Family XII;D_5__Exiguobacterium	11.27146381	0.000787076	0.001700085	0.042502126	0.000116618	0.00494506

Table S3. List of fungal taxa with significant differential abundance according to Kruskal-Wallis test

Taxa	Test-Statistic	P	FDR_P	Bonferroni_P	Endophytes mean	Epiphytes_ mean
k__Fungi;p__Basidiomycota;c__Tremellomycetes;o__Tremellales;f__Bulleribasidiaceae;g__Dioszegia	43.29204919	4.71E-11	9.90E-10	1.08E-09	0.000223633	0.014467867
k__Fungi;p__Basidiomycota;c__Tremellomycetes;o__Filobasidiales;f__Filobasidiaceae;g__Filobasidium	41.657613	1.09E-10	9.90E-10	2.50E-09	0.000269173	0.097417499
k__Fungi;p__Basidiomycota;c__Tremellomycetes;o__Tremellales;f__Bulleribasidiaceae;g__Vishniacozyma	41.28864878	1.31E-10	9.90E-10	3.02E-09	0.000160061	0.047828967
k__Fungi;p__Basidiomycota;c__Microbotryomycetes;o__Sporidiobolales;f__Sporidiobolaceae;g__Sporobolomyces	40.63092502	1.84E-10	9.90E-10	4.23E-09	0.000928789	0.092253084
k__Fungi;p__Basidiomycota;c__Agaricomycetes;o__Russulales;f__Russulaceae;g__Russula	40.32310309	2.15E-10	9.90E-10	4.95E-09	0.043177342	0.000290174
k__Fungi;p__Ascomycota;c__Dothideomycetes;o__Capnodiales;f__Cladosporiaceae;g__Cladosporium	39.76363636	2.87E-10	1.10E-09	6.59E-09	0.009474092	0.110059429
k__Fungi;p__Ascomycota;c__Dothideomycetes;o__Capnodiales;f__Mycosphaerellaceae;g__Mycosphaerella	38.68168393	4.99E-10	1.64E-09	1.15E-08	0.004987015	0.043398603
k__Fungi;p__Ascomycota;c__Dothideomycetes;o__Capnodiales;f__Mycosphaerellaceae;g__Zymoseptoria	37.91425943	7.39E-10	2.13E-09	1.70E-08	0	0.008099713
k__Fungi;p__Ascomycota;c__Sordariomycetes;o__Hypocreales;f__Ophiocordycipitaceae;g__Purpureocillium	35.58162162	2.45E-09	6.25E-09	5.63E-08	0.006458926	0
k__Fungi;p__Ascomycota;c__Dothideomycetes;o__Pleosporales;f__Pleosporaceae;g__Alternaria	34.29322858	4.74E-09	1.09E-08	1.09E-07	0.752765972	0.486943099
k__Fungi;p__Basidiomycota;c__Tremellomycetes;o__Tremellales;f__unidentified;g__unidentified	29.07059421	6.98E-08	1.46E-07	1.61E-06	0	0.003523891
k__Fungi;p__Ascomycota;c__Dothideomycetes;o__Pleosporales;f__Pleosporaceae;g__Stemphylium	27.75016835	1.38E-07	2.65E-07	3.17E-06	0.11681416	0.03321945
k__Fungi;p__Ascomycota;c__Dothideomycetes;o__Dothideales;f__Aureobasidiaceae;g__Aureobasidium	19.60850067	9.50E-06	1.68E-05	0.000218604	0.000657126	0.009309661
k__Fungi;p__unidentified;c__unidentified;o__unidentified;f__unidentified;g__unidentified	17.3905061	3.04E-05	5.00E-05	0.000699987	0.003305782	0.009831748
k__Fungi;p__Ascomycota;c__Saccharomycetes;o__Saccharomycetales;f__Debaryomycetaceae;g__Millerozyma	10.48493894	0.001203516	0.001845391	0.027680867	0	0.004462454

Table S4. Summary of Shannon index data

Shannon	Group1	Group2	Group1 mean	Group1 std	Group2 mean	Group2 std	t stat	*p*-value
Fungi	Endophytes	Epiphytes	7.066360839	0.13910637	7.720490151	0.222032593	-12.73013486	0.001
Bacteria	Endophytes	Epiphytes	5.863038023	0.41121461	6.507979267	0.646402543	-4.124119569	0.001
